# Identification of dominant *FOXE3* and *PAX6* mutations in patients with congenital cataract and aniridia

**Published:** 2010-08-22

**Authors:** Dominique Brémond-Gignac, Pierre Bitoun, Linda M. Reis, Henri Copin, Jeffrey C. Murray, Elena V. Semina

**Affiliations:** 1 Department of Pediatric Ophthalmology, St Victor University Hospital of Amiens, INSERM UMRS968, Vision Institute, Paris VI University, Picardie Jules Verne University, Amiens, France; 2 Department of Genetics, Jean Verdier Paris-Nord University Hospital, APHP, Bondy, France; 3 Department of Pediatrics and Children’s Research Institute at the Medical College of Wisconsin and Children’s Hospital of Wisconsin, Milwaukee, WI; 4 Department of Cytogenetics and Reproduction Biology, CGO University Hospital of Amiens, Picardie Jules Verne University, Amiens, France; 5 Department of Pediatrics, The University of Iowa, Iowa City, IA; 6 Department of Cell Biology, Neurobiology and Anatomy at the Medical College of Wisconsin, Milwaukee, WI

## Abstract

**Purpose:**

Aniridia and congenital cataract represent rare but severe developmental ocular conditions. We examined 33 probands from France for mutations in several transcription factors associated with these phenotypes, the forkhead box E3 (*FOXE3*), paired box gene 6 (*PAX6*), paired-like homeodomain transcription factor 2 (*PITX2*), and paired-like homeodomain transcription factor 3 (PITX3) genes.

**Methods:**

Out of 33 probands, 27 were affected with congenital cataract while the remaining six were affected with aniridia (with or without cataract). The coding regions of *FOXE3, PAX6, PITX2,* and *PITX3* were examined by direct DNA sequencing of gene-specific PCR products.

**Results:**

A novel dominant mutation at the stop codon of *FOXE3,* c.959G>C (p.X320SerextX72), was identified in a patient with congenital cataract. Another novel *FOXE3* sequence change, c.571–579dup (p.Tyr191_Pro193dup), was identified in a patient with aniridia, mild lens opacities, and some additional ocular defects; this patient was also found to carry a nonsense mutation in *PAX6*. *PAX6* mutations were identified in two additional probands with aniridia and cataracts. None of the observed sequence alterations were found in normal controls. No mutations were identified in *PITX2* or *PITX3.*

**Conclusions:**

The p.X320SerextX72 mutation is only the fourth *FOXE3* allele associated with a dominant phenotype since the majority of *FOXE3* mutations appear to be recessive with no phenotype observed in heterozygous carriers. The encoded protein is predicted to contain a complete normal sequence followed by seventy-two erroneous amino acids; the position and effect of this mutation are similar to two of the previously reported dominant changes, suggesting a common mechanism for dominant alleles. The p.Tyr191_Pro193dup is predicted to result in an in-frame duplication of three amino acids; however, the contribution of this mutation to the phenotype is unclear since the affected patient also carries a nonsense mutation in *PAX6* which acts upstream of *FOXE3* in the molecular pathway. The identified *PAX6* mutations correspond to the two most commonly observed mutant alleles and demonstrate phenotypes that are consistent with the previously reported spectrum.

## Introduction

Aniridia and congenital cataract represent rare but severe ocular conditions reflecting abnormal development of the anterior segment of the eye. Aniridia is characterized by the congenital absence of the iris and is most commonly inherited as an autosomal-dominant disorder. Aniridia is a panocular disease often associated with additional ocular symptoms such as visual impairment and nystagmus [1]. Congenital cataract is an opacification of the ocular lens present at birth. Congenital cataract is usually hereditary and can be transmitted as a dominant or a recessive trait. Congenital cataracts are frequently accompanied by other ocular defects, particularly anterior segment anomalies including corneal limbal deficiency and glaucoma with goniodysgenesis, but also posterior segment anomalies such as foveal hypoplasia [2]. The complexity of anterior segment phenotypes is likely due to close interaction of corresponding tissues during development and/or sharing of genetic factors that underlie their proper differentiation. Mutations in transcription factors that play active roles in directing normal embryonic developmental patterning were shown to result in a spectrum of anterior segment anomalies including aniridia and cataracts.

Mutations in forkhead box E3 (*FOXE3*), a forkhead transcription factor expressed in the lens which is located at chromosome 1p32, have been associated with both recessive and dominant ocular disease. Dominant mutations in *FOXE3* have been seen in patients with congenital cataract and/or anterior segment disease; three dominant disease-causing mutations have been reported to date [3-5]. Meanwhile, recessive mutations in *FOXE3* have been identified in eight families with aphakia, microphthalmia, and sclerocornea as the principal phenotype [5-8]. Obligate carriers in all recessive families were unaffected with the exception of one parent who was reported to have unilateral ocular ‘cloudiness’ [7].

Paired box gene 6 (*PAX6*) is a transcriptional regulator located at chromosome 11p13. Mutations or deletions of *PAX6* were first identified in patients with aniridia [9,10]. While aniridia remains the predominant phenotype associated with *PAX6* mutations [11], related ocular phenotypes including anterior segment malformations such as Peters’s anomaly [12], keratitis [13], foveal hypoplasia [14], congenital cataract [15], and optic nerve defects [16] were also shown to be associated with *PAX6* deficiency. Nonocular defects include abnormal brain structure, central auditory function, and olfactory bulb development [17-19]. Two patients with Gillespie-like syndrome (iris hypoplasia/aniridia, tremor/ataxia, and learning disability/mental retardation) were also found to have mutations in *PAX6* [20,21]. In addition, WAGR syndrome (Wilms tumor, Aniridia, Genitourinary anomalies, and mental Retardation) is associated with a contiguous gene deletion which includes *PAX6* and the WT1 gene (*WT1*) [22]. A recent review of *PAX6* mutations in the Human PAX6 Mutation Database  [23] found that mutations which lead to a premature termination codon are generally associated with aniridia while missense mutations typically result in other related ocular phenotypes [11]. This review also identified four CpG dinucleotides which are mutation hotspots accounting for 21% of mutations in the database and all resulting in aniridia [11].

Paired-like homeodomain transcription factor 2 (*PITX2*) is a homeodomain transcription factor at chromosome 4q25. Mutations in *PITX2* were first reported in patients with Axenfeld-Rieger syndrome [24]. Subsequently, the associated phenotypes have been expanded to include partial aniridia [25], Peters’ anomaly [26], iris hypoplasia/iridogoniodysgenesis syndrome [27,28], and ring dermoid of the cornea [29]. A wide range of mutations have been reported including missense, nonsense, splice-site, and intragenic deletions/duplications, along with whole gene deletions and translocations upstream of *PITX2;* the majority of these mutations result in Axenfeld-Rieger syndrome [30].

Mutations in paired-like homeodomain transcription factor 3 (*PITX3*), another homeodomain transcription factor located at chromosome 10q25, are associated with autosomal dominant congenital cataract with or without anterior segment dysgenesis [31]. A recurrent 17-bp insertion (c.657_673dup17) in the COOH-terminal region in the gene is the most common mutation, resulting primarily in congenital posterior polar cataract along with highly variable anterior segment mesenchymal dysgenesis (ASMD) in some individuals [31-35]. Two additional heterozygous mutations associated with isolated cataract have been reported [31,32,36]; one of these mutations, c.650delG, was also seen in homozygous form in one family, resulting in severe microphthalmia with neurologic deficits [36].

In this study, we screened thirty-three unrelated patients affected with congenital cataract and/or aniridia for mutations in *FOXE3, PAX6, PITX2,* and *PITX3.* Causative mutations were identified in *FOXE3* and *PAX6* but not *PITX2* or *PITX3*.

## Methods

The human studies were approved by Paris 7 University Hospital and the Institutional Review Boards of the Children’s Hospital of Wisconsin and the University of Iowa Hospitals and Clinics and informed consent was obtained for every subject. We screened a population of 33 probands from France: 27 were affected with congenital cataract while the remaining six were affected with aniridia (with or without cataract).

Gene-specific products were generated using the previously reported primers and conditions for *FOXE3* [3], *PAX6* [37], *PITX2* [24], and *PITX3* [31]. Briefly, PCR was conducted in 30 μl reactions containing 40 ng DNA, Biolase Reaction Buffer (Bioline, Taunton, MA), 0.25 mmol/l each dNTP, 1.5 units Biolase DNA polymerase (Bioline) and 0.2 micromol/l of each oligodeoxynucleotide primer.  Cycling profile included one cycle of 94 °C for 5 min followed by 30 cycles of 94 °C for 45 s, 55-60 °C (please see Table 1 for annealing temperature and other details) for 45 s, and 72 °C for 45 s, and one cycle of 72 °C for 10 min. The coding regions of *FOXE3, PAX6, PITX2,* and *PITX3* were examined by direct DNA sequencing of PCR products, as previously described [38]. Briefly, PCR products were sequenced in both directions using Big Dye Terminator v3.1 (Applied Biosystems, Foster City, CA) using a 3730XL DNA Analyzer (Applied Biosystems). Chromatograms were examined manually and all initially identified changes were confirmed by additional independent PCR and sequencing experiments. One hundred eighty two samples from healthy individuals of Caucasian decent were examined for the changes observed in *FOXE3* and *PAX6* using SSCP (single strand conformational polymorphism) analysis along with the corresponding patient samples as positive controls. In addition to this, the previously reported *FOXE3* normal variation data [7] and *PAX6* variation summarized in the Human PAX6 Mutation Database [23] were used for comparisons.

**Table 1:** PCR primers and conditions.

**Set**	**Forward primer**	**Reverse primer**	**Gene region**	**Annealing temperature (°C)**	**Product size (bp)**
***FOXE3* primers [**3**]**					
1	tgtccatataaagcgggtcg	atgtacgagtagggcggctt	Exon 1 (N-terminus)	56	298
2	ttctctggcttccctgccct	tcggtgatgaagcggtagat	Exon 1 (N-terminus, forkhead domain)	57	271
3	aagccgccctactcgtacat	tcgttgagcgtgagattgtg	Exon 1 (Forkhead domain)	56	170
4	ttcatcaccgaacgctttgc	aggaagctgccgttgtcgaa	Exon 1 (Forkhead domain)	56	185
5	aagggcaactactggacgct	tagctccggctgcaggttca	Exon 1 (Forkhead domain, C-terminus)	55	267
6	tctgttcagcgtcgacagc	acaggtcgcacaggtgcct	Exon 1 (C-terminus)	55	352
***PAX6* primers [**37**]**					
1	ctcatttcccgctctggttc	aagagtgtgggtgaggaagt	Exon 1	60	197
2	ttatctctcactctccagcc	aagcgagaagaaagaagcgg	Exon 2	60	276
3	tcagagagcccatcgacgtat	ctgtttgtgggttttgagcc	Exon 3	60	193
4	ttgggagttcaggcctacct	gaagtcccagaaagaccaga	Exon 4	60	153
5	cctcttcactctgctctctt	atgaagagagggcgttgaga	Exon 5	60	257
5a	tgaaagtatcatcatatttgtag	gggaagtggacagaaaacca	Exon 5a	55	237
6	gtggttttctgtccacttcc	aggagagagcattgggctta	Exon 6	60	299
7	caggagacactaccatttgg	atgcacatatggagagctgc	Exon 7	60	252
8	gggaatgttttggtgaggct	caaagggccctggctaaatt	Exon 8	60	371
9	gtagttctggcacaatatgg	gtactctgtacaagcacctc	Exon 9	60	206
10	gtagacacagtgctaacctg	cccggagcaaacaggtttaa	Exon 10	60	243
11	ttaaacctgtttgctccggg	ttatgcaggccaccaccagc	Exon 11	60	208
12	gctgtgtgatgtgttcctca	tgcagcctgcagaaacagtg	Exon 12	60	227
13	catgtctgtttctcaaaggga	gaacaattaacttttgctggcc	Exon 13	55	957
***PITX2* primers [**24**]**					
1a	ttggctcctaagtgcccc	ccagactcgcattatctcac	Exon 1a	56	596
1b	cttgacacttctctgtcagg	aagcgggaatgtctgcagg	Exon 1b	56	667
2	tagtctcatctgagccctgc	cactggcgatttggttctga	Exon 2	56	282
3	acgcctctctccgcacgt	ttcttgcgctttcgcccga	Exon 3	56	258
4	cagctcttccacggcttct	ttctctcctggtctacttgg	Exon 4	56	374
5a	gtaatctgcactgtggcatc	ctgtgggtgcggctcaca	Exon 5	56	627
5b	ctgagactgaaagcaaagca	ctcccatgaaataaaacacattt	Exon 5	56	787
***PITX3* primers [**31**]**					
1	cctggtctgccataaagtga	attctcgacctgttcccaag	Exon 1	55	343
2	acgcagccccagctttac	aagccagcgcatattctcc	Exon 2	55	395
3	gtgcaggacataacagcttc	gagcagaggctggaggttg	Exon 3	55	528
4a	ctctagccacctcatctcg	aggcataagggcaggacac	Exon 5	55	524
4b	agacctttccattcgccttc	agtcaaaatgaccccagtcc	Exon 5	55	483

## Results

**Figure 1:**
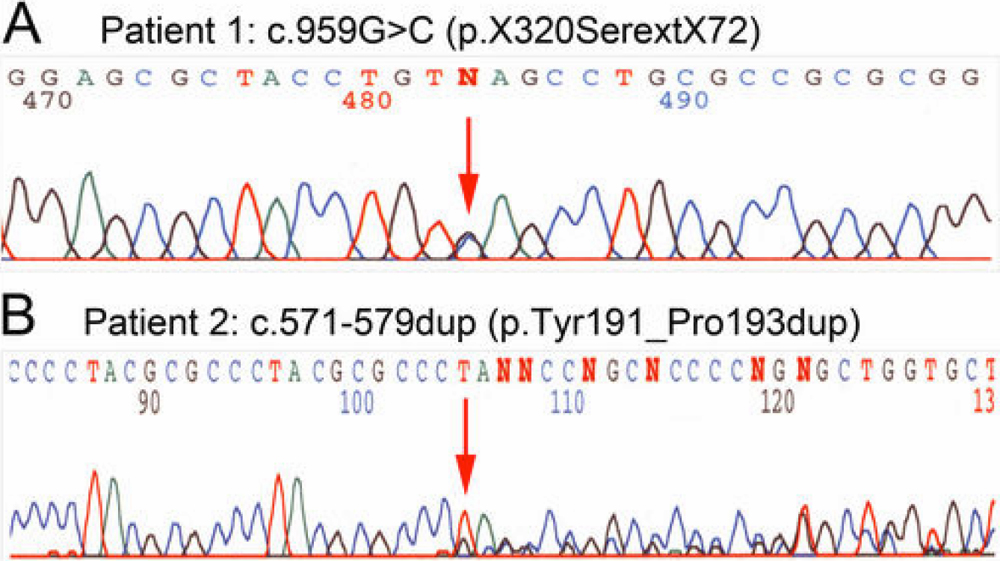
Novel *FOXE3* mutations. Fragments of DNA sequence trace chromatograms corresponding to the c.959G>C (p.X320SerextX73) mutation in Patient 1(**A**) and the c.571–579dup (p.Tyr191_Pro193dup) variant in Patient 2 (**B**). Mutation sites indicated with arrows.

**Figure 2:**
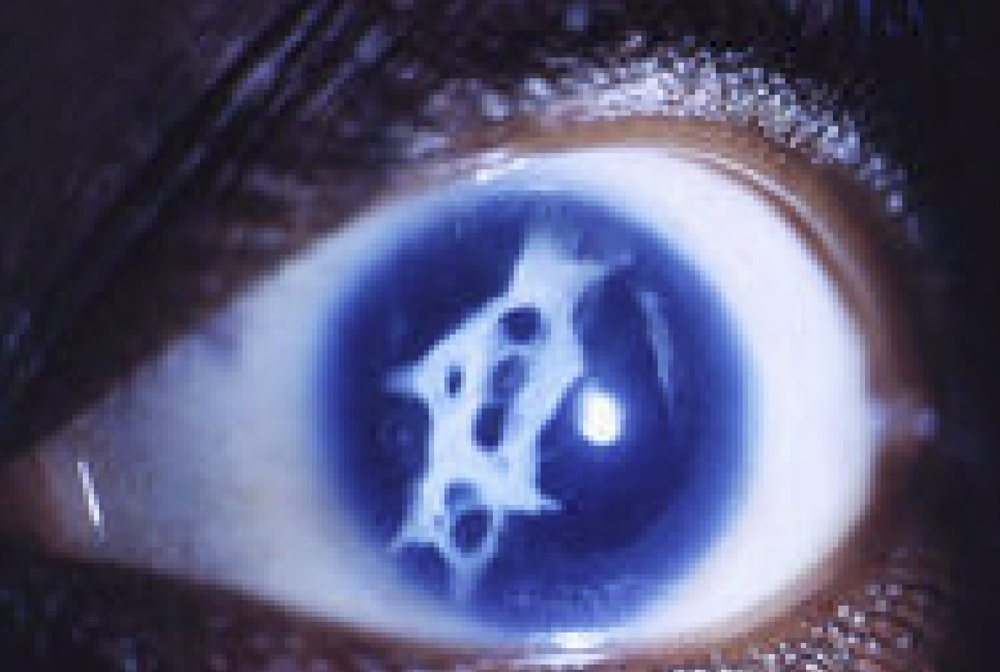
Patient photograph. Photographic image of the eye of Patient 2.

**Figure 3:**
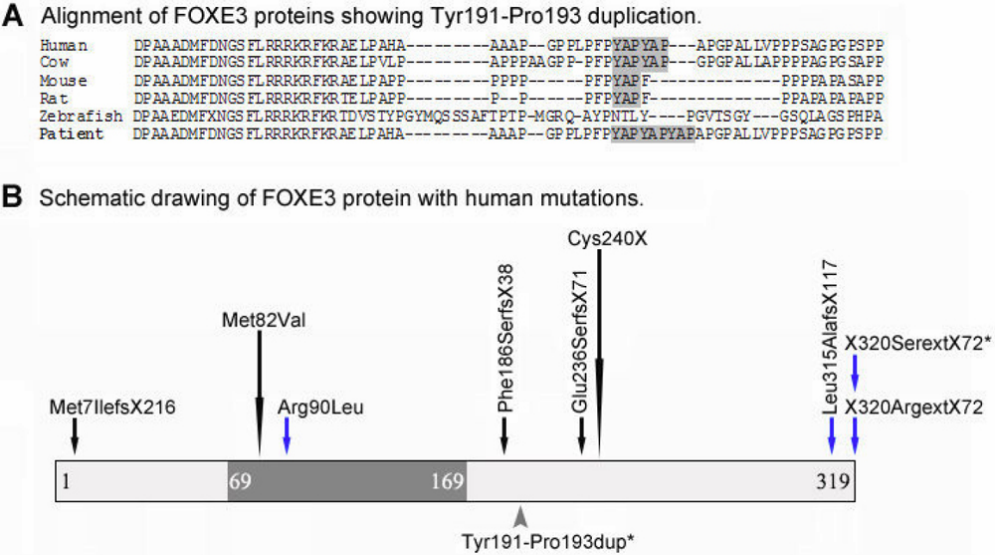
Summary of *FOXE3* mutations. **A**: Alignment of FOXE3 proteins showing region of p.Tyr191-Pro193 duplication. Please note two YAP amino acid motifs present in normal human and cow FOXE3 proteins and an additional insertion of this motif identified in Patient 2. **B**: Schematic drawing of FOXE3 protein with human mutations. Forkhead domain is shown in dark gray. Mutations resulting in recessive phenotypes are indicated with black arrows while mutations causing dominant disease- with blue arrows. The variant identified in Patient 2 is indicated with a gray arrowhead below the protein. Recurrent mutations are denoted with extended arrows. Positions of mutations identified in this study are shown with asterisks.

Screening of *FOXE3* identified a mutation in the stop codon of *FOXE3,* c.959G>C (p.X320SerextX72), in Patient 1 with congenital cataract (Figure 1A). There was no family history of ocular disease and no other family members were available for testing. The mutation results in a change of the termination codon into serine leading to the addition of 72 erroneous amino acids to the end of the protein. No other changes in *FOXE3* were identified in this patient. A second sequence alteration in *FOXE3* was identified in Patient 2 affected with aniridia, corneal limbal insufficiency, nystagmus, severe axile myopia, mild lens opacities, and development of a fibrous posterior capsular reaction after lens surgery (Figure 2). This *FOXE3* variant, c.571–579dup (p.Tyr191_Pro193dup), results in an in-frame duplication of 3 amino acids (Figure 1B and Figure 3A). The same patient was later found to also carry a *PAX6* mutation (see below). the parents of Patient 2 appear to be unaffected but were not available for testing. Neither of the identified *FOXE3* variants was seen in 182 Caucasian control individuals nor the previously reported 332 controls of mixed ethnicity [7].

Mutations in *PAX6* were identified in three unrelated probands with aniridia and cataract. Patient 2 (also carrying *FOXE3* mutation, please see above) was found to have a c.718C>T (p.Arg240X) mutation in *PAX6*. Patient 3, affected with aniridia and congenital cataract, was found to carry the same mutation. Finally, Patient 4, with aniridia and mild cataract, was found to carry a c.607C>T (p.Arg203X) mutation. The mother of Patient 4 also has aniridia and cataract but was not available for testing. Both of these mutations were previously reported in patients with aniridia (The Human PAX6 Mutation Database) [11]. No mutations in *PITX2* or *PITX3* were identified in this group of patients.

## Discussion

Mutation analysis of DNA obtained from patients affected with congenital cataracts (27) and aniridia with or without cataracts (6) identified two novel variants in *FOXE3*. One of these variants, p.X320SerextX72, apparently represents a causative mutation responsible for a phenotype of dominant congenital cataract, while the other variant, p.Tyr191_Pro193dup, is of unknown significance. Mutations in *FOXE3* appear to explain ~4% of congenital cataract cases in this population.

The p.X320SerextX72 mutation in *FOXE3* is only the fourth mutant allele associated with a dominant phenotype since the majority of *FOXE3* mutations appear to be recessive with no phenotype observed in heterozygous carriers (Figure 3B) [3-8]. The location and the predicted effect of this mutation are consistent with two of the previously described dominant mutations in *FOXE3*: p.Leu315AlafsX117 [3] and p.X320ArgextX72 [5] (Figure 3B). All three mutations are predicted to result in the addition of numerous erroneous amino acids to the most distant end of the FOXE3 protein. Two of these mutations, p.X320SerextX72 (reported here) and p.X320ArgextX72 [5], affect the stop codon and therefore are predicted to retain the entire normal FOXE3 sequence. The similarity between these mutations suggests a possible common mechanism for *FOXE3* dominant alleles. An additional dominant mutation which affects the DNA-binding domain of *FOXE3* has also been reported (p.Arg90Leu [4]).

The congenital cataract phenotype observed in the patient carrying the p.X320SerextX72 allele (Patient 1) is consistent with other dominant *FOXE3* mutations. The initially reported p.Leu315AlafsX117 mutation was found in a patient and her mother with cataracts and posterior embryotoxon; the proband was also affected with myopia [3]. The p.X320ArgextX72 mutation was identified in a family with a highly variable phenotype: the proband was affected with bilateral microphthalmia with aphakia and sclerocornea in the right eye and Peters’s anomaly and congenital cataract in the left while her mother and uncle had bilateral early onset cataract (age of diagnosis not given) with iris colobomas also seen in the uncle. A maternal aunt had a cataract extraction in her 20s and the maternal grandmother had a cataract extraction in her 40s. All of the above family members were found to carry the mutation while an unaffected maternal uncle did not carry the mutation [5]. Finally, the p.Arg90Leu dominant mutation affecting the DNA-binding domain of *FOXE3* was associated with Peters’ anomaly and glaucoma, but not cataract [4]. There was a family history of the disease consistent with dominant inheritance but no other family members were available for testing. In general, the phenotype associated with dominant mutations is milder than the recessive *FOXE3* phenotype (microphthalmia with aphakia in many cases), with the exception of the proband reported by Iseri et al. [5].

The effect of the p.Tyr191_Pro193dup mutation in *FOXE3* is unclear since Patient 2 also carries the nonsense mutation in *PAX6*. The patient does demonstrate mild anterior segment dysgenesis and lens opacities, but these features are not inconsistent with the *PAX6* mutation [11]. Since the identified *PAX6* mutation has been previously reported as causative in many families and since *Pax6* has been shown to act upstream of *Foxe3* in the ocular pathway [39], the phenotype of this patient is apparently caused by the *PAX6* deficiency. The effect of the p.Tyr191_Pro193dup mutation, if any, is most likely masked by this earlier molecular defect. It is also possible that this variant does make a contribution to the observed phenotype that is difficult to dissect in the presence of the *PAX6* mutation, or that it represents a very rare polymorphism. This case emphasizes the importance of the exclusion of mutations in major known genes in mutation screens to ensure accurate identification of the causative allele.

The identified mutations in *PAX6* are consistent with the associated phenotypes. These mutations, c.718C>T (p.Arg240X) and c.607C>T (p.Arg203X), are two of the most common mutations, both affecting a CpG mutational hotspot [11]. Of the 31 patients with the p.Arg240X mutation reported in the Human PAX6 Mutation Database, 26 have isolated aniridia while the remaining five have associated ocular anomalies (four with cataract). Of the 23 patients with the p.Arg203X mutation in the database, 18 are reported to have isolated aniridia and the remaining five have associated ocular anomalies (one with cataract).

The absence of *PITX2* mutations in examined patients is not surprising given its limited contribution to aniridia and a strong association with syndromic versus isolated anterior segment phenotypes. As for *PITX3*, which is linked primarily with cataracts with or without anterior segment defects, our findings suggest that it is not a major cause of congenital cataract.
